# Influence of in vitro test conditions on the Biomechanical properties of degenerated human lateral menisci

**DOI:** 10.1186/s13018-025-06151-x

**Published:** 2025-08-06

**Authors:** Michael Seidenstuecker, Luisa de Roy, Max F. Weiske, O. Piquet, Graciosa Quelhas Teixeira, Hermann O. Mayr, Bianca Riedel, Andreas M. Seitz

**Affiliations:** 1 G.E.R.N. Research Center for Tissue Replacement, Regeneration & Neogenesis, Department of Orthopedics and Trauma Surgery, Faculty of Medicine, Medical Center-Albert- Ludwigs-University of Freiburg, Albert-Ludwigs-University of Freiburg, Hugstetter Straße 55, 79106 Freiburg, Germany; 2 Institute of Orthopaedic Research and Biomechanics, Center of Trauma Research Ulm, Ulm University Medical Center, Helmholtzstr. 14, 89081 Ulm, Germany

**Keywords:** Meniscus, Osteoarthritis, Mechanical examination, Instantaneous modulus, Elastic modulus, Fixation methods, Sulfated glycosaminoglycan, Collagen

## Abstract

The biomechanical properties of degenerated meniscal tissue are increasingly being studied in the context of osteoarthritis research. Spatial indentation testing using a multiaxial testing machine allows non-destructive characterization of viscoelastic properties. However, in vitro testing conditions can significantly influence the results. The purpose of this round robin study was to evaluate the effects of different fixation methods and laboratory environments on the viscoelastic properties of degenerated lateral menisci. Spatial normal indentation tests were performed on nine degenerated human lateral menisci in two laboratories using a multiaxial testing machine. Key parameters, including the maximum applied force (P_max_), instantaneous modulus (IM), and elastic modulus (E_t10_), were analyzed across different meniscus regions. Significant differences in the IM, E_t10_, and P_max_ were observed between the laboratories, highlighting the influence of testing conditions on biomechanical results. The results indicated that variations in fixation methods, environmental conditions, and freeze-thaw cycles significantly affect the elastic and viscoelastic properties of meniscal tissue. Unphysiological strains in the inner region of the menisci suggested that strain-controlled indentation may be preferable to distance-controlled testing. These results underscore the importance of standardizing in vitro conditions for meaningful comparisons with the existing literature.

## Introduction

Knee osteoarthritis (OA) is a prevalent condition affecting millions globally [1], including 37% of persons aged 60 years and older [2], with multiple knee joint tissues implicated in its development. Furthermore, it is more common in women than in men [3]. The prevalence of OA is expected to increase with the aging populations in the U.S [4]. and Europe [5]. The meniscus plays a crucial role in load distribution and shock absorption, and its biomechanical dysfunction may accelerate OA progression [6, 7]. Meniscal impairment results from traumatic injuries and degeneration driven by microdamage accumulation, inflammation, and aging. Recently, increasing attention has been given to tissue alterations and the molecular mechanisms underlying meniscal injury and degeneration [8]. The biomechanical properties of degenerated meniscal tissue are of increasing interest in the context of OA research [9–11]. In contrast to biomechanical testing of extracted samples under confined or unconfined conditions, spatial indentation mapping allows for non-destructive mechanical characterization of the complex, wedge-shaped meniscus [10, 12]. However, when testing viscoelastic, biological tissues, the in-vitro test conditions are very likely to affect the outcome measures [11, 13]. Neither the U.S. Food and Drug Administration nor the American Society for Testing and Materials has published guidelines for the mechanical characterization of meniscal tissue. In their 2024 systematic review, Schwer et al. derived recommendations for mechanical testing based on a quantitative analysis of the reproducibility and reliability of the reviewed studies [14]. These can be used as a guide for future investigations and provide a basis for standardization and reporting guidelines. Moreover, the C4Bio initiative [15] was recently launched to achieve a common consensus on test protocols for material characterization of biological tissues, which could facilitate the development of standards for meniscus testing and reporting recommendations. On the basis of these prior works [14, 15], we derived the hypothesis that the in vitro testing conditions significantly influence the biomechanical parameters of lateral human menisci. Therefore, the aim of this study was to investigate the influence of different fixation methods and related laboratory environments on the elastic and viscoelastic properties of the same degenerated lateral menisci [10, 11]. Moreover, we aimed to assess the structure-function relationship in these menisci by adding biochemical quantifications.

## Materials and methods

### Study design

After IRB approval (IRB 305/10 Albert-Ludwigs University Freiburg) spatial indentation stress relaxation tests of nine degenerated lateral human menisci (70 ± 9 years; 44% female, 56% male) were performed in two independent laboratories using a multiaxial testing machine (Mach-1 v500css; Biomomentum Inc.). For this purpose, indentation stress relaxation tests were performed with thickness measurements at the lateral menisci to calculate the instantaneous modulus (IM), the modulus after a relaxation time of 10 s (E_t10_), and the maximum applied load (P_max_). Using the Mach-1 to map the IM of the menisci offers the advantage of non-destructive testing, unlike other approaches (e.g., punching out cylindrical samples). This method also allows for measurements at multiple locations with different sample fixation types, enabling the identification of possible differences. The degeneration state of the menisci was assessed by determining the water, sulfated glycosaminoglycan (sGAG), and collagen contents in both laboratories and correlated with the biomechanical data. Statistical and correlation analyses were performed to evaluate the results.

### Biomechanical tests

#### Stress relaxation tests

**Fig. 1:**
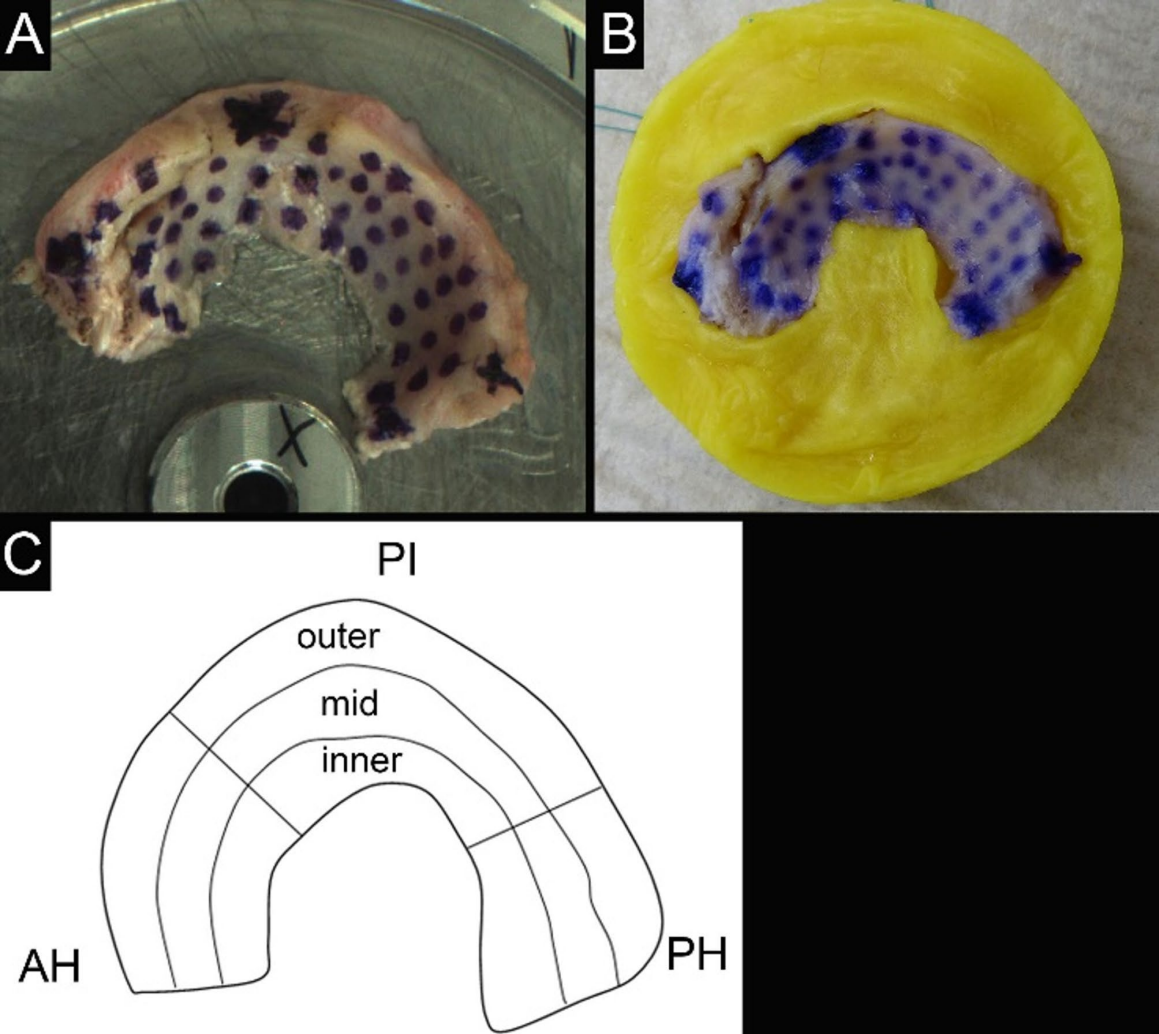
(**A**) Direct gluing of the meniscus to the specimen holder (Lab A); (**B**) PMMA customized confinement cast for each meniscus (Lab B); and (**C**) schematic representation of nine evaluated meniscus regions: anterior horn (AH); pars intermedia (PI); and posterior horn (PH)

The lateral menisci were harvested from nine patients who were initially indicated for a total knee replacement surgery. The tissues were tested in a fresh condition at Laboratory A (Lab A, Freiburg) using a multiaxial testing machine equipped with a multiaxial load cell (70 N; MA23X, ATI Industrial Automation, Apex, NC, USA). For tissue fixation, the menisci were directly glued on the specimen holder using fully removable adhesive (Fig. 1A) [9]. The non-residue removability was confirmed in pretests utilizing histological analyses. To ensure repeatability of the spatial mappings, both, the measurement points were marked directly on the meniscus surface using a tissue marker (Fig. 1A) and the indentation maps of each meniscus were exported by Lab A and re-imported in the software of the testing machine by Lab B (Ulm). The indentation parameters for both test runs were as follows: indenter diameter = 1 mm, indentation depth = 0.2 mm, indentation velocity = 0.2 mm/s, relaxation time = 10 s [9, 11]. After the first test, the menisci were frozen and shipped at − 30 °C to Lab B. After thawing the menisci were embedded in a custom-made polymethylmethacrylate (PMMA) cast to confine the meniscus along its circumference (Fig. 1B) as previously described [11]. During all tests, the samples were kept moist with saline solution. P_max_, as well as the IM representing the initial elastic response and the E_t10_ representing the initial viscous response of the meniscus tissue were determined. The IM was evaluated according to Hayes et al. [16] using the following equation for normal indentation:

$$IM\, = \,\frac{P}{H} \cdot \frac{{1 - \,{\vartheta ^2}}}{{2ak \cdot \left( {\frac{a}{h}\,\vartheta } \right)}}$$

Where IM is the instantaneous modulus; P is the load; H is the depth of indentation; a is the radius of the contact region; ϑ is the Poisson´s ratio (assumed 0.5 for soft tissues); k is the correction factor dependent on a/h and ϑ; and h is the sample thickness.

#### Thickness measurements

The thickness of the menisci was determined after the second test run using a needle technique, as described by Jurvelin et al. [17]. For this measurement, the indenter was replaced with a cannula (Sterican 18G; B. Braun, Melsungen, Germany). To prevent bending and potential inaccuracies in thickness measurements, the needle was replaced after each measurement point. The testing parameters were set as follows:

- Contact criterion: 0.1 N (indenter–sample).
- Stage velocity: 0.5 mm/s.
- Stage repositioning: 2× load resolution.
- Termination criterion: 5 N (to protect the load cell).

The same scan grid as used in the indentation test was applied. In this method, the needle’s displacement between the initial contact (0.1 N) with the meniscus tissue and the break-off contact (5 N) with the metallic base was recorded and analyzed using standard software of the multiaxial testing machine. The thickness measurement was adjusted using the cosine of the indentation angle, following the approach by Sim et al. [12] and Veronesi et al. [18]. The correction angle was automatically determined for all normal indentation positions.

### Biochemical characterization

The meniscus body was divided into three anatomical regions: anterior horn (AH), pars intermedia (PI), and posterior horn (PH), with a further subdivision in an inner, middle, and outer zone, leading in total to nine distinct regions (Fig. 1C). Cylindrical samples with a diameter of 3.8 mm were collected from each region. Then, their wet weight was determined using a precision scale (AC120S, Sartorius AG, Göttingen, Germany). Consecutively the samples were lyophilized (Labconco Freezone 2.5, Labconco, Kansas City, MA, USA), dry weight and their water content calculated. Subsequently, the samples were liquefied with proteinase K and their sGAG and DNA contents were determined with the dimethyl-methylene blue (DMMB)-Assay [19]. The soluble collagen content was determined for the AH, PI and PH regions following an established protocol [20] for Lab B and on the determination of hydroxyproline in Lab A [21].

### Statistical analyses

All data were tested for normality using the Shapiro-Wilk test. Since data were normally distributed, results are presented as mean ± standard deviation. Statistical analysis was performed using one-way ANOVA followed by Tukey’s post hoc test. Differences in P_max_, IM, and E_t10_ between Lab A and Lab B were assessed separately for all regions. A significance level of *p* < 0.05 was considered statistically significant. All analyses were conducted using Origin 2023 Professional SR1 (OriginLab, Northampton, MA, USA) and GraphPad Prism 10.2.2 (GraphPad Software Inc., Boston, MA, USA).

## Results

For our study, nine lateral menisci from four women and five men were used. The average age was 70 ± 9 years. First, the fresh menisci were measured in Lab A, where the mapping coordinates were also created, and then sent together with the fresh frozen menisci to Lab B.

### Mechanical properties

**Fig. 2:**
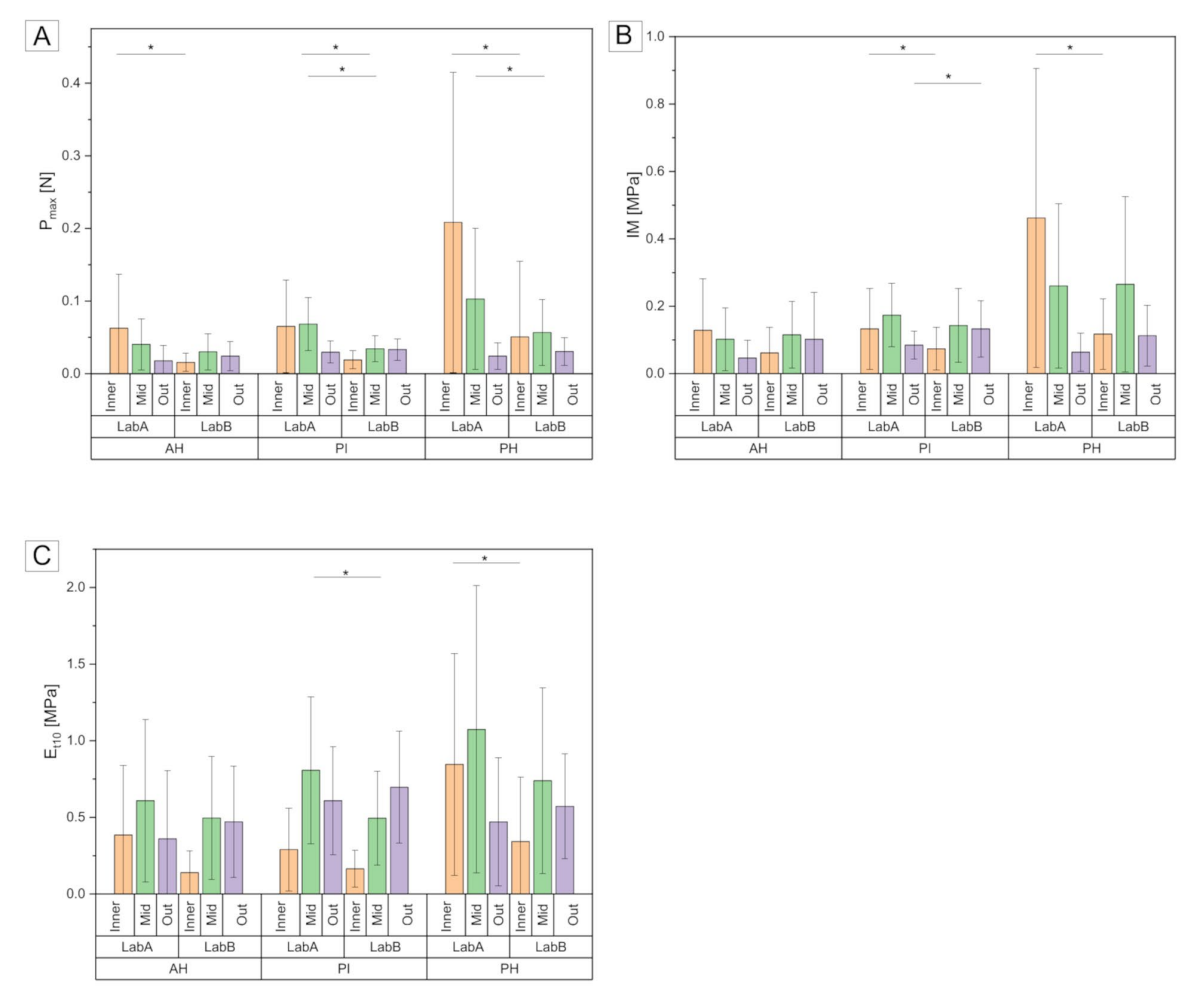
(**A**) Maximum applied force (P_max_) in N, (**B**) instantaneous modulus (IM) in MPa, and (**C**) relaxation modulus (E_t10_) in MPa obtained during the indentation measurements in both laboratories. Anatomical subdivision into the previously assigned nine regions (anterior horn (AH); pars intermedia (PI), and posterior horn (PH) and further subdivision into the inner (Inner), middle (Mid), and outer (Out) region). *n* = 9; non-parametric statistics; *p < 0.05; indentation parameters for lab A and B: indenter diameter = 1 mm, indentation depth = 0.2 mm, indentation velocity = 0.2 mm/s, relaxation time = 10 s, upper force limit = 5 N (to protect the load cell)

At the inner and middle meniscus zones, statistically lower values for the P_max_ (*p* < 0.05) were assessed in Lab B when comparing to those of Lab A, except for the AH middle zone (Fig. 2A). The P_max_ of the outer zones at the AH, PI, and PH was by tendency higher in Lab B compared to Lab A. Analyzing the elastic IM, significantly lower values were found in the inner and middle PI zones and in the inner PH zone in Lab B (*p* < 0.05) (Fig. 2B). In the outer PI and PH zones, the IM was statistically higher in Lab B (*p* < 0.05) compared to Lab A. No differences for the IM were found for the AH region. Statistically lower values for the viscoelastic E_t10_ were assessed in Lab B both, at the inner and middle zones of the AH, PI, and PH (*p* < 0.05) (Fig. 2C). In both test environments (Lab A, Lab B) the highest P_max_, IM, and E_t10_ values were assessed in the PH region of the lateral menisci. All outer zones displayed higher E_t10_ values in Lab B.

**Fig. 3:**
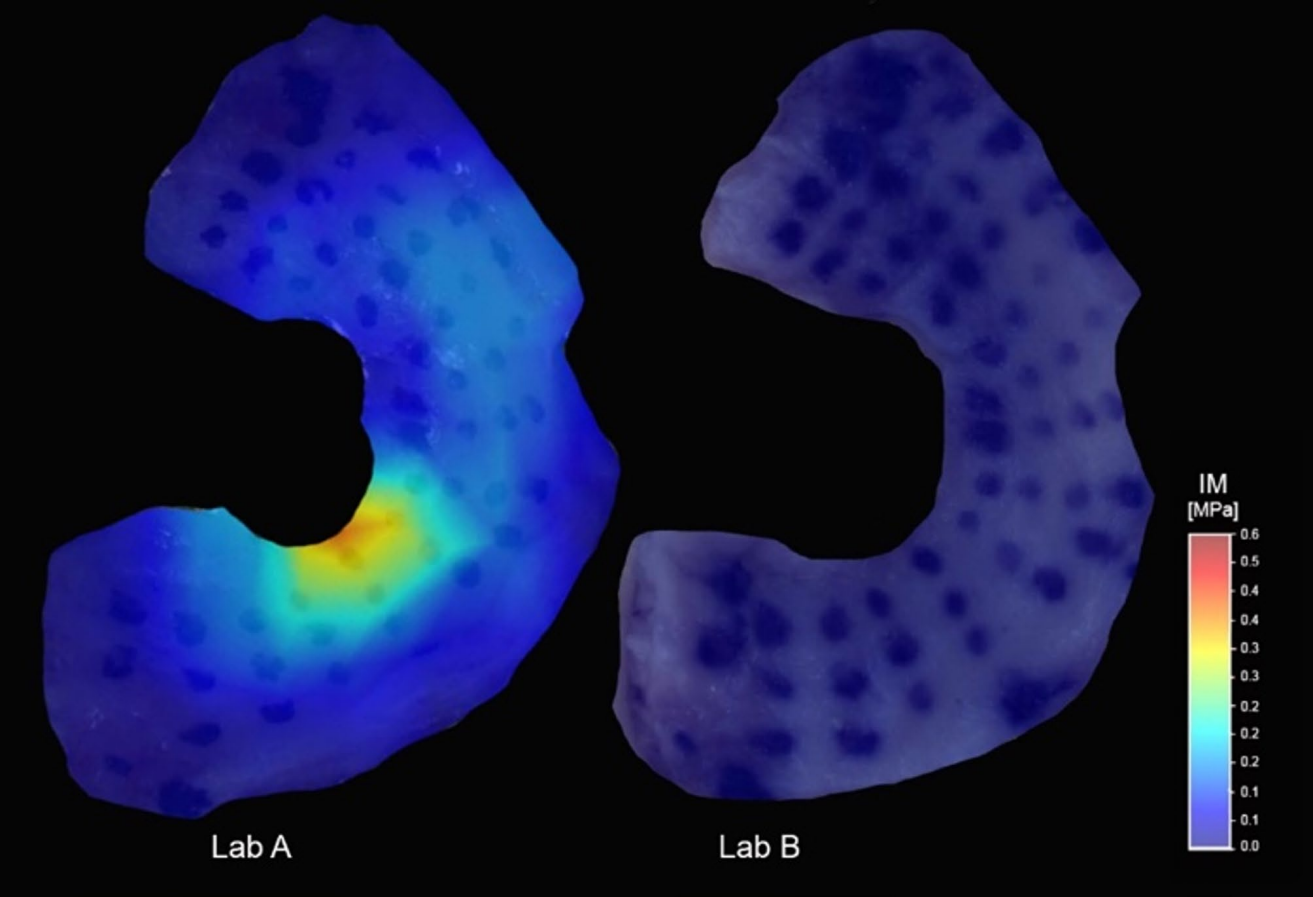
Exemplary mapping of the IM for both fixation methods. Lab **A**: Fixation with fully removable adhesive on the sample holder; Lab **B**: sample-specific PMMA substrate

The measured values for P_max_ were in a similar range (0–0.7 N) in both laboratories (Fig. 3). However, the samples measured by Lab A displayed higher values particularly in the inner area.

**Fig. 4:**
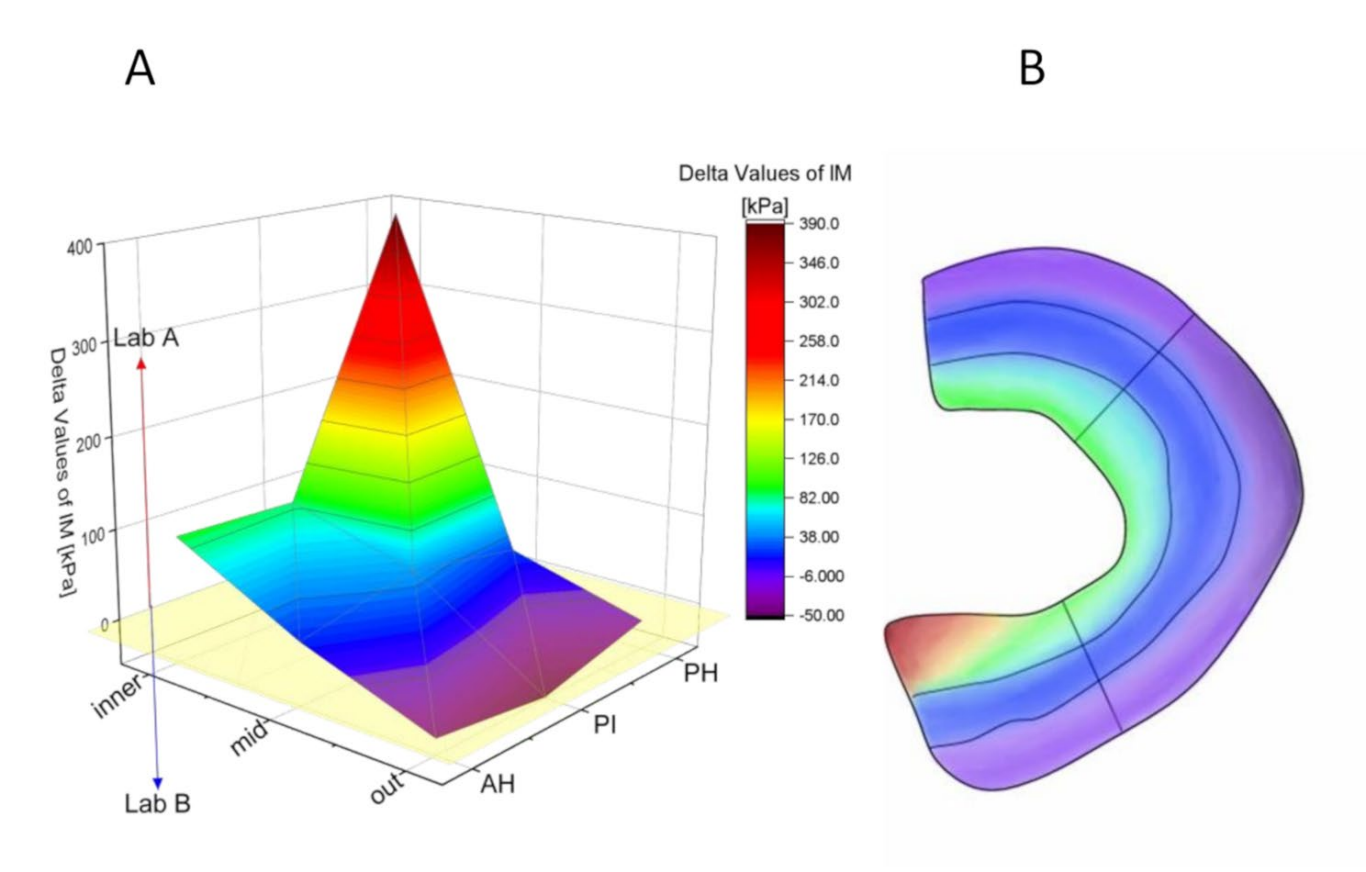
(**A**) Comparison of mean differences (Delta Values = (IM value Lab A)– (IM value Lab B)) of IM measurements in kPa in each meniscus region; (**B**) Quantitative reflection of the measurement differences to the nine different zones

In Lab A the inner zones were more loaded while in Lab B the outer zones experienced relatively more loading. These distinct differences were further analyzed by subtracting each according measurements to determine a qualitative map (Fig. 4). Starting from the yellow area, mainly all values related to the outer regions of the meniscus (in purple) were higher for Lab B. The middle area (dark blue) was in a similar range in both laboratories, being slightly higher for Lab A, while in the inner area (green to red) the values were clearly higher for Lab A.

### Biochemical properties

#### Water content

The water content was determined in both laboratories, with a mean value of 82.0 ± 2.3% in Lab A and 76.7 ± 2.9% in Lab B (Table 1).

**Table 1:** Mean values of the water content in the three meniscus regions, determined in both laboratories

Meniscus compartment	Mean water content [%]	
	Lab A	Lab B
AH	83.0 ± 4.1	78.1 ± 1.8
PI	79.4 ± 2.8	77.4 ± 2.2
PH	83.6 ± 4.2	74.5 ± 1.8

#### sGAG

**Fig. 5:**
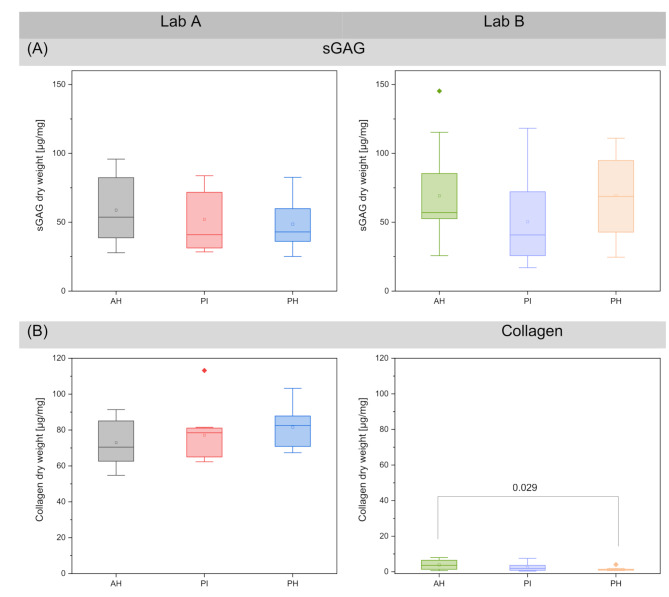
Boxplots indicating max, min, 75% and 25% percentiles and median values and of the (**A**) sGAG and (**B**) collagen dry weight; *n* = 9; Tukey posthoc test, non-parametric

The sGAG dry weight was determined in both laboratories and indicated comparable results: In detail, at the AH sGAG, determined in Lab B was 18% higher than in Lab A. The sGAG levels for the PI did not differ between the two laboratories, whereas for the PH a 42% higher value was measured in Lab B than in Lab A (Fig. 5A). No significant difference in the sGAG dry weight between the meniscal compartments was observed (*p* > 0.05).

#### Collagen

At Laboratory A, the collagen dry weight varies between 75 and 83 µg/mg, while at Laboratory B, they ranged between 3 and 4 µg/mg. When percentages are calculated, both laboratories are in the same range, 3–5%. For the AH, Lab B determined a value of 5.2 ± 3.6% (*p* = 0.0001) of the collagen content as that determined by Lab A. For the PI, the collagen content determined by Lab B was 3.1 ± 2.7% (*p* < 0.001) of the value as that determined by laboratory A, and for the PH, 1.7 ± 1.2% (*p* < 0.001) of the value determined by Lab A (Fig. 5B). No significant difference was found for the collagen values determined by Lab A. The collagen content determined by Lab B indicated a significant difference between the AH and PH (*p* = 0.029).

## Discussion

The aim of this study was to investigate the influence of different fixation methods and related laboratory environments on the elastic and viscoelastic properties of the same degenerated lateral menisci. Moreover, the degeneration state of the meniscus samples was identified by means of biochemical quantifications. The fixation method in Lab A was based on directly gluing the menisci to the specimen holder, while in Lab B a confining cast, made of PMMA was used. The results displayed higher IM values in the inner zones of the glued meniscus fixation, while the PMMA embedded menisci indicated higher IM values in the outer zones, thus corroborating our hypothesis. In addition, differences in biochemical parameters were observed which could be attributed to the additional freezing/thawing cycle as well as to different measurement techniques for the collagen content [22, 23].

### Mechanical properties

This study indicated that both, the initial elastic and viscoelastic properties are influenced by the meniscus fixation method during indentation stress relaxation testing. At the inner zone, where the meniscus is thin, the defined indentation depth of 0.2 mm could have led to very high, unphysiologic strains in the meniscus tissue which led to higher P_max_ and IM values (particularly for the fixation method applied in Lab A) compared to the outer zones. A similar result is seen by Pordzik et al. [10]– there, the thinner outer areas of the meniscus were rated above average in terms of IM as well. Seitz et al. [11] came to similar conclusions regarding mildly degenerated menisci. They used the same fixation method as Lab B. Fixation B confined the circumference without the need to glue the sample to the older, explaining the lower P_max_, IM, and E_t10_ values in the inner zones and slightly higher values in the outer zones. A cast would be preferable to gluing to the test holder, but it should be much less rigid than the PMMA cast used in the present study. In our opinion, a silicone cast, mimicking the confinement ability of the joint capsule might be the best choice. Furthermore, strain-controlled indentation testing would be superior for spatial indentation testing of viscoelastic tissues compared to distance-controlled testing. In this way, unphysiologically high strains could also be avoided, particularly in the case of anatomically thin structures such as the inner zone of the lateral meniscus. Furthermore, each meniscus underwent a freeze-thaw cycle and was tested twice, which is known to decrease the intrinsic compressive resistance of meniscal tissue [4], potentially impacting the biomechanical outcome parameters. Ekiert et al. [24] demonstrated a correlation between the number of freeze-thaw (F/T) cycles and the tensile properties of subcutaneous deep frontalis tendon fascicle bundles, and that as the number of F/T cycles increase, the tensile properties decrease. Following one F/T cycle, the measured values were already 8.5% less, after two cycles 14% smaller, and increasing to 19% after three cycles. This influence can be expected to be transferred to other soft tissues in a similar manner as described above, but with different characteristics, which would explain the differences in the E_t10_ readings between Labs A and B.

### Biochemical properties

The water content tended to decrease slightly after freezing in the measurements of Lab B. Other authors similarly showed that the water content may depend on the degree of degeneration [25, 26]. Son et al. [27] determined the water content of OA menisci to be similar to ours at 79.6%, as did Morejon et al. [28] with 76.8% (Table 2). Finally, while there was a slight difference in the water content between the two laboratories, no significant difference was found.

**Table 2:** Mean water content as a percentage of the menisci of human knee joints; values from the literature in comparison with the results of the present study

Author		Water content (%)		Comments
	*n*_total_ lat/med	Lateral	Medial	
Herwig et al. [26]	17/0	77.1		Increased water content as degeneration progressed
Bursac et al. [29]	33/25	75.4	77.9	Healthy allograft group
Son et al. [27]	13/7	79.6	78.0	OA menisci
Danso et al. [30]	13/13	79	79	
Seitz el al. [31]	25/25	72.2	73.3	
Lewis et al. [13]	9/9	59.1	58.1	
Warnecke et al. [25]	24/0	76.3		Increased water content as degeneration progressed
Morejon et al. [28]	3/5	76.8		
Katsuragawa et al. [32]		76.5	75.2	
Nishimuta [33]				
Seitz et al. [11]	36/36	75.0	76.4	Severely degenerated
Present study	9/0	82.0 76.7		Fresh (Lab A) Fresh frozen (Lab B)

Like a previous study on lateral degenerated menisci [5], no zone-specific dependencies were found for the collagen or sGAG content. For both laboratories, the measured values for sGAG were in the same measurement range with no significant difference, since similar assays based on the DMMB assay were used (Table 3). The sGAG content was determined by Aggad et al. [34] also using a DMMB assay. Unfortunately, the meniscus was not divided into compartments, but is given as a total value of 3.2 ± 0.48 µg/mg. These values differ by a factor of 16–20 from our measurements (Labs A and B). However, this may be due to the fact that Aggad et al. used a slightly different method. Instead of freeze-drying the samples, they digested them with papain at 65 °C for 6 h and subsequently incubated them with chondroitin lyase for 30 min before adding them to the DMMB complex. Rothrauff et al. [35] obtained slightly lower dry matter values for sGAG than ours, but they were still in the same range. They also performed a DMMB assay. Sanchez-Adams et al. [36] obtained a similar range as those in the present study with slightly lower values, which can be explained by using a chondroitinase assay to determine the sGAG content. In the study by Schwartz et al. [37], the tibial region exhibited a significantly higher sGAG value compared to the femoral and core regions. This finding is consistent with the results of Murphy et al. [38], who reported significant regional variation in sGAG content, with the highest values observed in the outer femoral and tibial regions. Spierings et al. [39] determined the following sGAG content: 41.4 ± 13.6 µg/mg in decellularized samples and 34.1 ± 14.2 µg/mg in native controls. Gouldin et al. [40] found that sGAG levels ranged from 1 to 15 µg/mg, depending on age and gender. They demonstrated that the proportion of sGAG increases with age.

**Table 3:** Overview of mean sGAG content in the present study compared to other studies

Author	sGAG content [µg/mg]			Comments
	AH	Pi	PH	
Aggad et al. [34]	3.2	3.2	3.2	Menisci not subdivided
Gouldin et al. [40]	1–15	1–15	1–15	Menisci not subdivided
Otani et al. [8]	18	25	30	Blyscan Glycosaminoglycan Assay Kit
Rothrauff et al. [35]	31.7	31.7	31.7	Menisci not subdivided into AH/PI/PH
Sanchez-Adams et al. [36]	9.1	12.0	38.8	
Spierings et al. [39]	34.1	34.1	34.1	Menisci not subdivided into AH/PI/PH
Nishimuta et al. [41]	7	7	7	Menisci not subdivided
Morejon et al. [28]	8.8	8.8	8.8	Menisci not subdivided
Nishimuta et al. [33]	161	161	161	Menisci not subdivided
Present study	58.8	52.0	48.6	Fresh (Lab A)
	69.3	50.5	69.3	Fresh frozen (Lab B)

When measuring collagen content, a difference was observed between the values obtained by the two laboratories in the present study (Table 4). This can be attributed since Lab A used the hydroxyproline content to determine the total collagen content, whereas Lab B used a Sircol collagen assay (SCA). Lareu et al. [42] demonstrated that the SCA can overestimate collagen content by a factor of 3–24 compared to the hydroxyproline method of determining collagen content. Aggad et al. [34] also determined the amount of hydroxyproline in their study and obtained a similar order of magnitude as Lab A. However, they distinguished between males and females as well as between lateral and medial menisci, finding 84.4 ± 7.2 µg/mg in lateral menisci of males and 79.3 ± 6.7 µg/mg in females. Gouldin et al. [40] investigated how collagen content varies with age and between women and men. They found values ranging from 30 to 90 µg/mg. Spierings et al. [39] measured a collagen content of 82.2 ± 4.8 µg/mg in native samples and 87.9 ± 7.7 µg/mg in decellularized samples.

**Table 4:** Overview of mean collagen content in the present study compared to other studies

Author	Collagen content [µg/mg]			Comments
	AH	Pi	PH	
Aggad et al. [34]	84.4 79.3	84.4 79.3	84.4 79.3	Males (not subdivided) Females (not subdivided)
Danzo et al. [30]	14	15	14	
Gouldin et al. [40]	30–90	30–90	30–90	Menisci not subdivided
Rothrauff et al. [35]	758	758	758	Menisci not subdivided
Spierings et al. [39]	82.2	82.2	82.2	Menisci not subdivided
Present study	72.9	77.1	81.6	Fresh (Lab A)
	3.8	2.4	1.4	Fresh frozen (Lab B)

### Clinical relevance

The findings presented in this study have potential clinical relevance, particularly in the context of orthopedic decision-making. Observed regional differences in the biomechanical and biochemical properties of the anterior, middle, and posterior zones of the human meniscus suggest that meniscal preservation or reconstruction strategies could be optimized based on the affected subregion [43]. For instance, if the posterior horn - often subject to higher mechanical loads, exhibits greater stiffness and lower sGAG content, this could influence surgical approaches. For example, it could lead to a preference for tailoring of repair techniques to preserve functionally critical zones [44, 45]. Furthermore, recognizing subregional variations in collagen and sGAG content can guide the development and positioning of meniscal scaffolds or implants to more closely resemble native tissue properties [46, 47]. Finally, the methods used in this study could serve as a basis for developing diagnostic or prognostic tools to assess early degenerative changes in specific meniscal regions. These tools would enable more targeted and personalized orthopedic interventions [48, 49].

### Limitations

Several factors must be taken into account when assessing the reliability of the results of this study. As with other experiments on ex vivo preparations, the investigations presented here cannot replicate the complex load behavior of the natural knee joint and the circumferential strains that occur during loading. Therefore, the indentation behavior determined here cannot be equated with that of an intact joint with menisci. The indentation properties are influenced by the surface layer and the locally defined area around the indenter. Nevertheless, indentation is more suitable for material characterization than other methods (e.g., punching and compression tests) because it allows us to image the entire meniscus and not just parts of it (punching out, which may have been shortened to make them plane-parallel). The current settings of the Mach 1 software do not allow the influence of the substrate to be minimized, especially when only distances can be entered. This results in thin areas of the meniscus being indented disproportionately more than thicker areas. In extreme cases, this can also lead to the substrate being measured, resulting in measurements that are too large. Furthermore, indentation mapping of the entire meniscus surface allows for high spatial resolution and non-destructive testing but leads to prolonged testing times with autolysis.

## Conclusions

This round robin study showed that different in vitro test conditions (fixation methods, environmental conditions, freeze-thaw cycle) are affecting both the initial elastic and viscoelastic as well as biochemical properties of degenerated human meniscus tissue. Therefore, caution is advised when comparing one’s own results with those described in the literature. This is most relevant if the results are to be used as a basis for later measurements. These results are valuable for researchers in the field of soft tissue biomechanics. They can be used to advance the standardization of soft tissue measurements, creating a uniform basis for all researchers. Additionally, readers should be better informed about how to interpret the obtained data.

## Data Availability

The raw data supporting the conclusions of this article will be made available by the authors, without undue reservation.
